# Lens Capsule microRNA Profiling Across the Primary Angle-Closure Disease Spectrum: Association with Anterior Segment Crowding

**DOI:** 10.3390/ijms27219556

**Published:** 2026-10-26

**Authors:** Hae Min Park, Hyun Soo Cho, Yeji Yeon, Min-Ji Cha, Jong Sub Lee, Soorack Ryu, Jooyoung Yoon, Yu Jeong Kim, Won June Lee

**Affiliations:** 1 Department of Ophthalmology, College of Medicine, Hanyang University, Seoul 04763, Republic of Koreahscho1204@hanyang.ac.kr (H.S.C.);; 2 Department of Ophthalmology, Hanyang University Seoul Hospital, Seoul 04763, Republic of Korea; 3 Department of Medicine, College of Medicine, Hanyang University, Seoul 04763, Republic of Korea; 4 Department of Ophthalmology, Hanyang University Guri Hospital, Guri 11923, Republic of Korea; 5 Hanyang Institute of Bioscience and Biotechnology, Hanyang University, Seoul 04763, Republic of Korea

**Keywords:** primary angle closure disease, microRNA, anterior lens capsule, anterior segment biometry, lens vault

## Abstract

We aimed to compare anterior lens capsule microRNA (miRNA) expression across the primary angle-closure disease (PACD) spectrum and a control group, and to examine associations between differentially expressed miRNAs and anterior segment biometric parameters. In this cross-sectional study, anterior lens capsule specimens were collected during cataract surgery from 27 eyes (seven controls, nine primary angle-closure suspect (PACS) eyes, six primary angle-closure (PAC) eyes, and five primary angle-closure glaucoma (PACG) eyes). The expression of 84 miRNAs was profiled, and the ΔCt values were compared across groups and correlated with anterior segment biometric parameters. Experimentally validated target genes of significant miRNAs were retrieved from miRTargetLink 2.0. miR-188-5p was the only miRNA that differed significantly between control and PACD eyes (control 11.27 ± 2.29 vs. PACD 8.21 ± 2.09; *p* = 0.003), with higher expression in PACS (*p* = 0.022) and PAC (*p* = 0.006). Higher miR-188-5p expression correlated with greater LV (r = −0.64, *p* = 0.033), narrower AOD500 (r = 0.50, *p* = 0.015), and shorter AXL (r = 0.41, *p* = 0.032). miR-146b-5p and miR-335-5p showed subgroup-specific differences and correlated with LV and AXL/AOD500, respectively, though these findings were exploratory. However, these findings did not remain significant after correction for multiple testing. Network analysis identified four experimentally validated targets of miR-188-5p (*GREM2*, *ITGA7*, *CEMIP*, *FGF5*) relevant to extracellular matrix turnover, hypoxia and inflammation. miR-188-5p was upregulated in the anterior lens capsule across the PACD spectrum and was associated with biometric hallmarks of a crowded anterior segment, suggesting its potential as a diagnostic or therapeutic candidate for PACD, pending validation in larger, functional studies.

## 1. Introduction

Primary angle-closure disease (PACD) is a spectrum in which appositional or synechial iridotrabecular contact obstructs aqueous outflow, raising intraocular pressure (IOP) and threatening the optic nerve. Following the International Society of Geographical and Epidemiological Ophthalmology (ISGEO) scheme, it is staged as primary angle-closure suspect (PACS; ≥180° of iridotrabecular contact with normal IOP and a healthy disc), primary angle closure (PAC; contact with raised IOP or peripheral anterior synechiae), and primary angle-closure glaucoma (PACG; with glaucomatous optic neuropathy) [1]. PACG is a leading cause of irreversible blindness and is concentrated in Asian populations [2,3]. The disease spectrum is of particular interest because it often progresses to bilateral blindness and is frequently detected late [4].

Its anatomical basis is well described: affected eyes show a crowded anterior segment, with a shallow anterior chamber, short axial length, and a thicker, more anteriorly positioned crystalline lens, and so on [5]. Yet, these biometric descriptions do not explain the molecular processes that operate within the lens and its surrounding tissues. Accumulating evidence suggests that the crowded anterior segment is also a site of inflammation and oxidative stress [6,7]. Previously, the aqueous humor of angle-closure eyes was reported to show elevated chemokines (CXCL8, CXCL9) in chronic PACG and pro-inflammatory cytokines (IL-6, IL-12, IL-15, IL-27, TNF-α) in acute attacks, together with higher VEGF, MCP-1, MMP-2 and TIMP-1 [7,8,9,10]. Aqueous TGF-β2 was also reported to be elevated with sustained IOP elevation [11]. Molecular markers, such as COL1A1, VEGF-B, VEGF-C and VEGFR2, were also differentially expressed in iris tissues of PACG eyes compared to those of POAG eyes [12]. Since the lens epithelium is also constantly exposed to this aqueous humor and vulnerable to oxidative damage, its fibrotic or inflammatory behavior may, therefore, serve as a window into that of the adjacent anterior segment [8].

The anterior lens capsule is removed during cataract surgery, and is a quite readily accessible tissue lined by lens epithelial cells (LECs). MicroRNAs (miRNAs)—small non-coding RNAs that post-transcriptionally regulate proliferation, extracellular matrix remodeling, epithelial–mesenchymal transition (EMT), and stress responses—are stable in ocular tissues and have emerged as candidate biomarkers of many eye diseases. miRNA expressions in the aqueous humor and lens capsules of glaucoma patients have been reported, and let-7 family members were suggested to regulate TGF-β2/Smad-driven fibrosis and EMT of LECs [13,14,15]. However, miRNAs in lens capsules have not been compared across the PACD spectrum, nor linked to the biometric features that characterize it.

Therefore, we compared anterior lens capsule miRNA expression patterns across the PACD spectrum (PACS, PAC and PACG) and a control group, and examined how differentially expressed miRNAs were associated with anterior segment biometric parameters. Candidate miRNAs were interpreted in light of their known biology to generate hypotheses about the pathophysiological and structural processes that may underlie PACD.

## 2. Results

### 2.1. Baseline Characteristics of the Study Group

A total of 27 eyes were analyzed: seven control eyes, nine with PACS, six with PAC, and five with PACG (Table 1). The four groups did not differ significantly in sex, age, cataract grade, spherical equivalent and CCT (all *p* > 0.05). However, AXL differed significantly among the four groups (*p* = 0.040), being shortest in the PACS group (21.96 ± 0.81 mm) and longest in controls (23.62 ± 1.15 mm).

### 2.2. Differential miRNA Expression Across Primary Angle-Closure Disease

Of the 84 miRNAs analyzed, only miR-188-5p differed significantly between controls and the PACD group (control 11.27 ± 2.29 vs. PACD 8.21 ± 2.09; *p* = 0.003), with a lower ΔCt (i.e., higher expression) in the PACD group. (Table 2) On post hoc pairwise testing, miR-188-5p expression was significantly higher in PACS (*p* = 0.022) and PAC (*p* = 0.006) than in controls, while a similar but non-significant trend was observed in PACG (*p* = 0.116).

In addition, five miRNAs—let-7b-5p, miR-146b-5p, miR-215-5p, miR-335-5p, and miR-504-5p—showed a significant difference in at least one pairwise subgroup comparison without reaching significance in the control-versus-PACD comparison. miR-146b-5p differed between control and PACS eyes, whereas miR-335-5p differed between PACS and PACG eyes. However, these pairwise findings were not supported by significant overall four-group or control-versus-PACD comparisons and should, therefore, be interpreted as exploratory findings requiring validation in larger cohorts. The full 84-miRNA dataset is provided in Supplementary Table S1.

Because miR-188-5p was the only miRNA differing significantly both across the four groups and between the control and PACD groups, it was included for further analysis. Additionally, miR-335-5p and 146b-5p were also retained together with miR-188-5p for the subsequent correlation analysis, because they showed significant differences in sub-group comparisons (PACS vs. PACG, and control vs. PACS, respectively). Because differences limited to certain disease stages may not appear when all PACD eyes are combined, these two miRNAs were also included in the correlation analysis as exploratory candidates.

### 2.3. Correlation Between miRNA Expression and Anterior Segment Biometry

Correlations between the three miRNAs and anterior segment biometric parameters are shown in Table 3. miR-188-5p ΔCt correlated positively with axial length (r = 0.41, *p* = 0.032) and AOD500 (r = 0.50, *p* = 0.015), and negatively with lens vault (r = −0.64, *p* = 0.033). miR-335-5p ΔCt correlated positively with axial length (r = 0.52, *p* = 0.005) and AOD500 (r = 0.43, *p* = 0.042). miR-146b-5p ΔCt correlated negatively with lens vault (r = −0.75, *p* = 0.008). No significant correlations were observed between any of the three miRNAs and maximum IOP, CCT, ACD, or lens thickness. After FDR correction, none of these correlations remained significant.

Because ΔCt is inversely related to expression, these findings indicate that higher expression of miR-188-5p was associated with a shorter axial length and lower AOD500 (i.e., narrower angle), as well as with greater lens vault. Lower expression of miR-335-5p was associated with a longer axial length and wider AOD500 (i.e., a more open angle). In contrast, higher expression of miR-146b-5p was associated with a greater lens vault.

### 2.4. Differential Expression of miRNA Between Acute Angle-Closure Attack and No Attack Within PACD Group

A total of 20 PACD eyes were analyzed, of which 10 eyes had a history of acute attack and 10 eyes did not. No miRNA showed a statistically significant difference in expression between the attack versus non-attack groups (all *p* > 0.05) (Supplementary Table S2).

### 2.5. Target Gene Network of miR-188-5p

**Figure 1:**
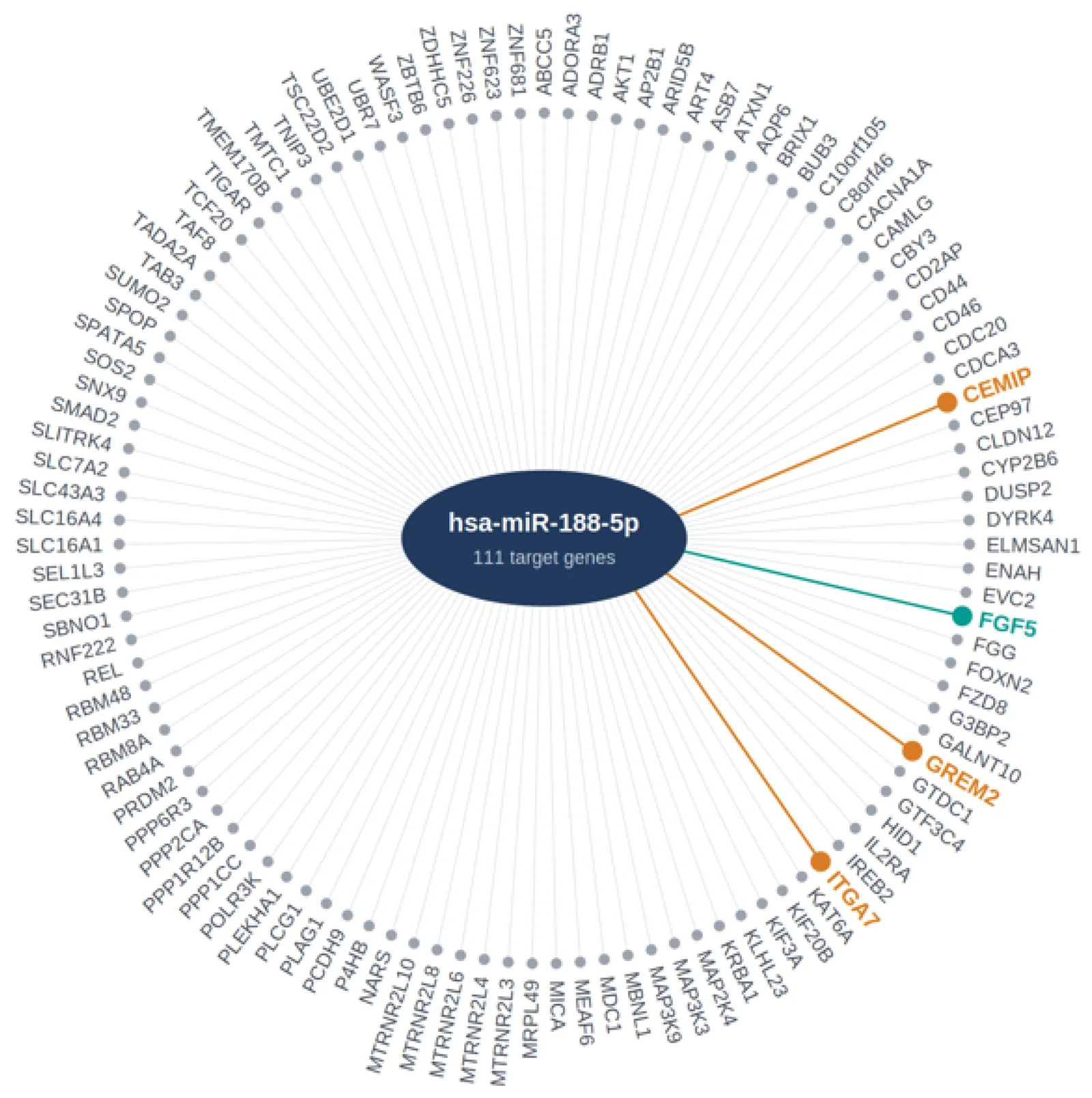
Target gene network of has-miR-188-5p. The 111 experimentally validated target genes were retrieved from miRTargetLink 2.0 using strong-evidence interactions only. 4 strongly validated target genes discussed in the text are highlighted: *CEMIP*, *GREM2*, *ITGA7* and *FGF5*.

Experimentally validated target genes of hsa-miR-188-5p—the miRNA that differed significantly across the PACD spectrum—were retrieved from miRTargetLink 2.0, yielding 111 target genes (Figure 1). Of these, four were classified as strongly validated interactions: *GREM2*, *ITGA7*, *CEMIP*, and *FGF5*. These four targets encode, respectively, a gremlin-family bone morphogenetic protein antagonist (*GREM2*), the α7β1 laminin-binding integrin (*ITGA7*), a hyaluronan-degrading enzyme (*CEMIP*; also known as KIAA1199/HYBID), and a fibroblast growth factor family ligand (*FGF5*).

## 3. Discussion

Among the 84 miRNAs studied, miR-188-5p was the only one that differed significantly between the control and PACD groups (*p* = 0.003), and was consistently upregulated in PACD compared to the control group. On post hoc pairwise testing, this upregulation reached significance for controls versus PACS (*p* = 0.022) and controls versus PAC (*p* = 0.006), with a concordant but non-significant trend for PACG (*p* = 0.116), most plausibly reflecting the small PACG subgroup (*n* = 5). Interestingly, the miRNA showed correlations with biometric hallmarks of the crowded anterior segment: higher expression of miR-188-5p was associated with a greater lens vault (r = −0.64 for ΔCt, *p* = 0.033), a narrower angle (AOD500; r = 0.50, *p* = 0.015) and a shorter axial length (r = 0.41, *p* = 0.032), recalling that a lower ΔCt denotes higher expression [5,16]. These associations suggest that miR-188-5p expression is related to anatomical features of PACD; however, because of the cross-sectional design and small sample size, the overexpressed miR-188-5p and its hypothetical associations with PACD should be regarded as speculative.

One proposed mechanism of PACD is that the crystalline lens becomes bulky and anteriorly positioned via age-related growth of lens fibers and, together with age-related zonular laxity, forward vaulting that shallows the anterior chamber [17]. In its pathogenesis, genome-wide studies have implicated contributions of ECM-related genes such as *COL11A1* (collagen X1) and apical cell-junction genes such as *PLEKHA7* as the most likely candidate susceptibility genes for PACG [18]. The study also reported that these genes were expressed in tissues that form the iridocorneal angle, including the iris, the zonule that suspends the lens, and the elastic fiber network of the trabecular meshwork (TM) [18]. In this context, miR-188-5p may be of interest because its principal reported role is to slow the breakdown of ECM and push cells toward growth and fibrosis. Previously, it was reported to directly repress matrix-degrading enzymes such as MMP-2 and MMP-13, so when miR-188-5p is highly expressed, ECM is cleared slower and accumulates in the tissue [19,20]. In hepatic stellate cells, it was also reported to restrain matrix breakdown while promoting fibroblast activation [19,21]. Our pathway analysis of miR-188-5p also corroborates these prior studies.

Network analysis identified four strongly experimentally validated targets of miR-188-5p—*GREM2*, *ITGA7*, *CEMIP*, and *FGF5*—whose functions map closely to the physiology of angle closure. *GREM2* encodes a gremlin-family antagonist of morphogenetic proteins (BMPs); in TM, gremlin-mediated BMP antagonism removes the physiological brake on TGF-β2 signaling, thereby increasing extracellular-matrix deposition and elevating intraocular pressure [22]. *CEMIP* (also termed KIAA1199 or HYBID) is a principal hyaluronan-degrading enzyme that drives matrix remodeling and fibrosis [23]. Because hyaluronan is a major constituent of juxtacanalicular outflow matrix, its turnover influences outflow resistance [24]. *ITGA7* encodes the major laminin binding integrin of muscle, which anchors cells to the basement membrane and transduces mechanical signals—a function pertinent to the ciliary muscle and to the mechano-sensing of mechanical load in anterior segment tissues [25,26,27]. In addition, *FGF5* is a member of the fibroblast growth factor family, and FGF signaling is the principal driver of lens epithelial proliferation and fiber differentiation, and, hence, of lens growth; miR-188-5p is reported to directly repress FGF5 [28,29]. Therefore, the target genes of miR-188-5p (*GREM2*, *CEMIP*, *ITGA7* and *FGF5*) are relevant in the composition, turnover or mechano-sensing of the outflow tract and angle matrix, as well as the growth of the crystalline lens, the structures important in PACD and acute angle-closure physiology.

In our cohort, higher miR-188-5p expression was associated with greater lens vault and shorter axial length, but not with lens thickness. This finding is consistent with the long-recognized anatomic configuration where lens vault rises as axial length shortens, which results in a large lens relative to a small eye [5,30,31]. Throughout life, equatorial lens epithelium adds secondary fibers via FGF signaling. Previous experimental studies reported that the epithelium proliferates at low FGF concentrations and differentiates at high concentrations [28,32]. The same epithelium is co-regulated by TGF-β/Smad signaling; in the normal lens, it is reported to control proliferation and differentiation, but when left unchecked, it drives the epithelial–mesenchymal transition (EMT) of lens epithelial cells that produces fibrotic cataract [33]. The FGF and TGF-β/Smad signals act interdependently, as they act on the same cells and their balance decides cell fate. Because FGF-driven ERK signaling was reported to be able to oppose TGF-β/Smad activity and steer epithelial cells toward orderly fiber differentiation instead of EMT [33,34], miR-188-5p may be relevant in this context via its validated target, *FGF5* [29]. At the same time, these FGF and TGF-β signals are previously reported regulators of scleral re-modeling and axial growth of the globe [35,36]. Although speculative, these observations raise the hypothesis that miR-188-5p-related signaling may be associated with a short axial length and lens–globe disproportion; our data, however, cannot establish any causal role. However, this postulation remains tentative, as *FGF5* is one of several lens FGFs and most references for the above hypothesis are derived chiefly from FGF1 or FGF2. However, as *FGF5* is one variant of the FGF family, miR-188-5p may have at least a partial influence on the above-described pathways regarding lens growth, cataract development and axial length, which all contribute to the lens–globe disproportion that underlies angle-closure pathophysiology.

Zonular weakness—which destabilizes the lens and predisposes to pupillary block and angle crowding—is another well-established mechanism in PACD [5,37]. However, this is difficult to reconcile with miR-188-5p’s validated targets as zonular weakening reflects proteolytic degradation of the fibrillin-1 microfibrillar scaffold, implying increased matrix turnover [37], whereas miR-188-5p represses the matrix-degrading enzymes MMP-2 and MMP-13, which would instead slow such degradation [20]. Therefore, the lens–globe disproportion mechanism described above is a more plausible explanation for the high lens vault of this study cohort and for the function of miR-188-5p in the context of PACD.

A more coherent—though still speculative—link would be with the trabecular meshwork. By repressing MMP-2 and MMP-13 and the hyaluronidase *CEMIP*, miR-188-5p could slow juxtacanalicular matrix turnover, which might, in turn, favor matrix accumulation and stiffening of the TM [20,23]. This would raise outflow resistance, along with the reduced MMP that would also lower outflow facility [24,38]. Loss of mechano-sensing integrin ITGA and gremlin family BMP antagonism (*GREM2*) may also contribute to this process via further impairing pressure-homeostatic remodeling of the TM [22,25,26,27]. Although this hypothesis cannot fully be supported by the current results, it might have some relevance to the observation that relieving mechanical angle crowding or PACD with controlled IOP does not always equate to ‘no progression’ [5,39,40].

Beyond matrix remodeling, miR-188-5p and its targets are also linked to hypoxic and inflammatory signaling. miR-188-5p is reported to be upregulated under ischemic conditions, where its silencing attenuates TNF- α, IL-1β and IL-6 production, and its repression of PTEN activates PI3K/AKT signaling, which increases HIF-1 α levels under hypoxia [21,41,42]. Among its targets, *CEMIP* is reported to be induced by hypoxia and inflammatory cytokines and generate pro-inflammatory hyaluronan fragments, whereas *GREM2* and FGF 5 limit inflammation and ischemic injury in non-ocular diseases. This implies that miR-188-5p may modulate anterior segment responses to hypoxic and inflammatory stress in both directions [43,44,45,46]. As several targets of miR-188-5p are also reported to participate in retinal ganglion cell (RGC) survival—via pathways such as PI3K/AKT and MAPK/JNK kinases—and amelioration of synaptic dysfunction in the central nervous system, it may also contribute to glaucomatous progression that is independent of IOP [47,48,49]. However, this hypothesis should be validated with future studies, and should be interpreted as a mere assumption based on previous study results.

The above-proposed pathophysiologic mechanisms require further validation; however, if confirmed in larger cohorts, miR-188-5p may be further explored as a candidate molecular marker associated with PACD. As lens extraction is an established treatment for PACD, capsular miR-188-5p collected at surgery could complement anterior segment biometry in identifying those at risk of PACD, persistent IOP elevation, or progression despite relief of angle crowding. Detection of similar signatures in more accessible samples, such as aqueous humor, would also aid in earlier identification of anatomically predisposed eyes or disease monitoring. Any therapeutic implication remains highly speculative at this stage, and would first require confirmation that the proposed pathways are operative in relevant ocular tissues [22,24,26,38,47,48,49,50].

Beyond miR-188-5p, miR-146-5p and miR335-5p also showed subgroup-specific differences (control vs. PACS; PACS vs. PACG, respectively), each correlating with distinct biometric parameters: lens vault for miR-146b-5p and axial length/AOD500 for miR-335-5p. The findings regarding miR-146-5p and miR335-5p, small subgroup sizes and a lack of significance in the overall comparisons should be regarded as exploratory rather than markers of disease stage. Nonetheless, the fact that the subgroup differences reached statistical significance despite small sample sizes implies that miR-146b-5p and miR-335-5p may still merit further investigation as candidate markers, independent of the specific correlation directions reported here.

This study has several limitations. The sample was small, particularly the PACG subgroup (*n* = 5), which limited statistical power and generalizability. Second, given the limited sample size, none of the associations remained statistically significant after correction for multiple testing. Although the observed correlations were moderate to strong in magnitude and directionally consistent with the anatomical features of PACD, they should be interpreted as hypothesis-generating. The effect sizes reported here may inform sample size calculations for future validation studies. The design was cross-sectional, retrospective, and single-center, including a single ethnicity, and, therefore, cannot establish causal, temporal, or progression-related relationships. miRNA expression was surveyed with a targeted 84-miRNA hypoxia panel rather than by genome-wide profiling; therefore, disease-relevant miRNAs outside this panel may have been missed, and our findings may not represent the entire capsule’s miRNome. In addition, the anterior lens capsule—though accessible and disease-relevant—is only a surrogate for the anterior-segment tissues (trabecular meshwork, zonule, retina) in which the proposed mechanisms would operate, so tissue-level inferences remain indirect. Finally, the target and pathway analyses were derived from interaction databases without functional validation with ocular cells or tissues. Furthermore, several of these associations—most notably the *FGF5*-related lens pathway—rest largely on studies of FGF1/FGF2; the proposed hypotheses should, therefore, be regarded as speculative, particularly since some of miR-188-5p’s predicted target effects are not concordant. Finally, as the anterior lens capsule can only be obtained during cataract surgery, it is, therefore, not suitable for routine screening or monitoring. Future studies should examine whether miR-188-5p and related miRNAs are similarly altered in more accessible samples, such as aqueous humor, or minimally invasive samples, such as tears or plasma, and whether their levels correspond to those of the lens capsule.

## 4. Methods

### 4.1. Study Design and Participants

This cross-sectional study included glaucoma patients and control patients who visited the Department of Ophthalmology of Hanyang University Seoul Hospital between April 2023 and December 2024. The institutional review board (IRB) of Hanyang University Seoul Hospital reviewed and approved the study protocol (IRB file No.: HYUH 2018-10-013-003, Approval date: 18 December 2018), and the protocol of this study adhered to the tenets of the Declaration of Helsinki. All study participants provided written informed consent to participate in this study.

This study recruited 29 patients who were scheduled for a routine cataract surgery: 9 PACS, 6 PAC, 7 PACG and 7 control patients were recruited and their medical charts were retrospectively reviewed. Two PACG patients were excluded from the final analysis due to erroneous miRNA analysis results. The inclusion criteria for the control group were an eye scheduled for cataract surgery with no previous history of intraocular surgery, no ocular disease other than cataract and no use of topical ocular medication. The inclusion criterion for the PACD group was a diagnosis of PACD in an eye scheduled for cataract surgery. The exclusion criteria for both groups were secondary angle closure of any cause, including neovascular, uveitic, traumatic or lens-induced glaucoma. Corneal or other ocular disorders precluding accurate anterior segment measurement or prior intraocular surgery other than glaucoma related procedures were also excluded. In addition, those with poor anterior segment data imaging quality or erroneous miRNA analysis results were not included in the final analysis. Furthermore, only one eye per participant was included in the final analysis.

Diagnosis of PACD was based on the International Society of Geographical and Epidemiological Ophthalmology (ISGEO) [1]. An occludable angle was defined as one in which the posterior trabecular meshwork was not visible on non-indentation gonioscopy for at least two quadrants in the primary position. PACS was defined as an occludable angle with a normal intraocular pressure (IOP) and no peripheral anterior synechiae (PAS) or glaucomatous optic neuropathy; PAC as an occludable angle with features of trabecular obstruction such as elevated IOP or PAS but without glaucomatous optic neuropathy; and PACG as the presence of glaucomatous optic neuropathy with a compatible visual field defect in an eye with an occludable angle. All gonioscopic examinations and diagnoses were performed by a single glaucoma specialist (W.J.L.) using non-indentation gonioscopy.

### 4.2. Ophthalmic Examination and Anterior Segment Biometry

All participants underwent a complete ophthalmic examination, including measurement of best-corrected visual acuity (BCVA), manifest refraction, slit-lamp biomicroscopy, IOP measurement by Goldmann applanation tonometry, gonioscopy, dilated fundus examination, and SS-OCT (DRI-OCT Triton; Topcon, Tokyo, Japan). Cataract severity was graded using the lens opacities classification system III (LOCS III) by 2 experienced ophthalmologists (Y.J.K and W.J.L) [51]. As posterior subcapsular opacity was rarely observed in this cohort, cataract grade was defined as the sum of the nuclear opalescence (NO) and cortical (C) scores. Additionally, participants in the PACD group also underwent stereo disc photography, red-free photography (EIDON confocal scanner; CenterVue, Pauda, Italy), and 30-2 perimetry using the Swedish interactive thresholding algorithm (Humphrey Field Analyzer II; Carl Zeiss Meditec, Jena, Germany). Maximum IOP (max IOP) was defined as the highest IOP recorded during the follow-up period.

Ocular biometry, including axial length (AXL) and lens thickness, was measured (ANTERION, Heidelberg Engineering Inc., Heidelberg, Germany). Central corneal thickness (CCT) was measured using an ultrasound pachymeter (Tomey SP-3000; Tomey Corporation, Nagoya, Japan) by the same technician, recording a mean of ten consecutive readings. Anterior segment parameters, including anterior chamber depth (ACD), angle opening distance at 500 µm from the scleral spur (AOD500), lens thickness (LT), and lens vault (LV), were obtained using anterior segment optical coherence tomography (ANTERION, Heidelberg Engineering Inc., Heidelberg, Germany). AOD500 was measured at the 0° and 180° meridians, and the mean of the two values was used for analysis. In cases where automated segmentation of the scleral spur or angle recess failed, the corresponding landmarks were manually identified and adjusted by two experienced examiners (H.M.P and W.J.L) using the device’s built-in caliper tool.

### 4.3. Lens Capsule Sample Collection

The anterior lens capsule was obtained as the central continuous curvilinear capsulorhexis specimen (approximately 5–6 mm in diameter) at the beginning of cataract surgery. An intact capsulorhexis was performed under a surgical microscope, avoiding contact with or damage to the iris and other intraocular structures. Each specimen was transferred to an RNase-free tube and immediately frozen at −80 °C until further processing. The lens capsule samples were collected by glaucoma (W.J.L.) and corneal specialists (Y.J.K).

### 4.4. Total RNA Extraction, Complementary DNA (cDNA) Synthesis, and Real-Time Polymerase Chain Reaction

Anterior lens capsule specimens were homogenized in QIAzol Lysis Reagent (Qiagen, Hilden, Germany), and total RNA, including small RNAs and miRNAs, was extracted using the miRNeasy Micro Kit (Qiagen) according to the manufacturer’s instructiosn. Extracted RNA samples were stored at −80 °C until further analysis. The quality of the extracted RNA was evaluated by measuring the OD260/280 ratio using a spectrophotometer (NanoDrop ND-2000; Thermo Fisher Scientific, Waltham, MA, USA). Complementary DNA (cDNA) was synthesized using the miRNCURY LNA RT Kit (Qiagen) according to the manufacturer’s protocol. miRNA expression was profiled using the miRCURY LNA miRNA Focus PCR Panel: Human Hypoxia Signaling (Qiagen), which contains assays for 84 miRNAs. This panel was selected a priori because IOP elevation in angle closure has been associated with ocular hypoxia. In addition, as hypoxia signaling is closely linked to inflammation and fibrosis, this panel also included miRNAs involved in inflammatory and extracellular matrix pathways. Quantitative real-time polymerase chain reaction (qRT-PCR) was performed using a QuantStudio 3 Real-Time PCR System (Thermo Fisher Scientific, Waltham, MA, USA) with SYBR Green-based detection according to the manufacturer’s instructions.

### 4.5. Normalization and Quantification of miRNA Expression

Normalization of miRNA expression, particularly in the absence of stable miRNA, was performed in reference to previously described research [52]. First, raw cycle threshold (Ct) values were obtained for each miRNA assay. Then, miRNA expression levels were normalized using U6, SNORD44, and SNORD49A as endogenous reference controls. miRNA expression levels were normalized using the geNorm algorithm with U6 snRNA (v2), SNORD44, and SNORD49A as predefined reference controls. Relative miRNA expression was quantified using the comparative 2^−ΔΔCt^ method, where ∆Ct = Ct (target gene) − Ct (reference gene) and fold changes were calculated relative to the control group [53,54].

### 4.6. Bioinformatics Analysis

Bioinformatics analysis was performed for miRNAs showing significant differential expression among the study groups. Experimentally validated target genes were retrieved from miRTargetLink 2.0 [55]. In this platform, interaction networks between miRNAs and target genes integrate experimentally validated targets curated from databases including miRTarBase and miRATBase. Interactions are stratified by the level of experimental support: “strong evidence” requires direct validation via low-throughput functional assays (such as dual-luciferase reporter assays, Western blotting, or qPCR), whereas “weak evidence” comprises high-throughput screening data (such as CLIP-seq or microarray profiling). To identify high-confidence functional effectors among the 111 experimentally supported targets, targets backed by strong experimental evidence were prioritized for detailed molecular mapping and mechanism formulation, while the full target set was mapped to illustrate the broader interactome.

### 4.7. Statistical Analysis

Continuous variables are presented as mean ± SD and categorical variables as *n* (%). Baseline homogeneity among the control, PACS, PAC, and PACG groups was assessed using the Kruskal–Wallis test for continuous variables and Fischer’s exact test for categorical variables. miRNA ΔCt values were compared across the four groups using the Kruskal–Wallis test, with Dunn’s test for post hoc pairwise comparisons, and between the control group and the pooled PAC spectrum group (PACS + PAC + PACG) using the Mann–Whitney U test. Correlations between differentially expressed miRNAs and anterior segment biometric parameters were evaluated using the Spearman correlation coefficient. To account for multiple comparisons, *p*-values were adjusted using the Benjamini–Hochberg false discovery rate (FDR) method for correlation analyses. Given the exploratory nature of this study, both raw and FDR-adjusted *p* values are reported. All statistical analyses were performed using R version 4.6.1 (R Foundation for Statistical Computing, Vienna, Austria), and a two-sided *p*-value < 0.05 was considered statistically significant.

## 5. Conclusions

In this exploratory, cross-sectional study, miR-188-5p was upregulated in the anterior lens capsule of eyes across the PACD spectrum and was associated with crowded-angle biometry, including greater lens vault, narrower angle and shorter axial length. These findings are hypothesis-generating and insufficient to establish a causal relationship; validation in larger, longitudinal studies is warranted.

## Figures and Tables

**Table 1:** Baseline characteristics of the study groups.

Characteristic	Control (*n* = 7)	PACS (*n* = 9)	PAC (*n* = 6)	PACG (*n* = 5)	*p*
Sex, male, *n* (%)	4 (57.1)	1 (11.1)	2 (33.3)	0 (0.0)	0.090
Age, years	71.7 ± 8.2	71.2 ± 7.7	68.2 ± 3.8	76.8 ± 5.8	0.251
Cataract grade	3.29 ± 1.50	1.78 ± 1.79	1.67 ± 1.97	2.50 ± 2.29	0.352
Spherical equivalent (D)	0.64 ± 0.57	1.36 ± 2.70	0.13 ± 0.41	0.85 ± 1.65	0.613
AXL (mm)	23.62 ± 1.15	21.96 ± 0.81	22.48 ± 1.12	23.08 ± 0.57	0.040 *
CCT (um)	537.3 ± 34.5	541.1 ± 21.8	554.2 ± 28.9	509.2 ± 33.5	0.177

Data are presented as mean ± SD for continuous variables or *n* (%) for continuous variables. * Significant at *p* < 0.05. PACS, primary angle-closure suspect; PAC, primary angle closure; PACG, primary angle-closure glaucoma; D, diopters; AXL, axial length; CCT, central corneal thickness.

**Table 2:** Differential expression (ΔCt) of selected miRNAs between control and PAC spectrum groups (PACS, PAC, PACG), and inter-group comparisons.

miRNA	Control (Group 1)	PACS (Group 2)	PAC (Group 3)	PACG (Group 4)	PACD (Group 2–4)	Among Groups	Groups 1 vs. 2~4	Groups 1 vs. 2	Groups 1 vs. 3	Groups 1 vs. 4	Groups 2 vs. 3	Groups 2 vs. 4	Groups 3 vs. 4
let-7b-5p	−0.34 ± 1.29	−1.44 ± 0.71	−1.34 ± 0.48	−0.87 ± 0.52	−1.27 ± 0.62	0.074	0.109	0.046 *	0.093	0.412	0.770	0.141	0.149
miR-146b-5p	6.91 ± 1.88	5.03 ± 1.03	5.59 ± 0.97	5.88 ± 0.85	5.41 ± 0.99	0.056	0.083	0.023 *	0.150	0.285	0.317	0.145	0.608
miR-188-5p	11.27 ± 2.29	8.34 ± 2.23	7.44 ± 1.63	8.91 ± 2.42	8.21 ± 2.09	0.022 *	0.003 *	0.022 *	0.006 *	0.116	0.414	0.664	0.259
miR-215-5p	5.72 ± 2.43	4.51 ± 1.00	5.49 ± 0.54	5.40 ± 1.27	5.03 ± 1.04	0.397	0.487	0.251	0.816	0.791	0.048 *	0.174	0.868
miR-335-5p	7.15 ± 3.16	4.42 ± 1.06	5.55 ± 1.09	6.03 ± 1.48	5.16 ± 1.32	0.065	0.151	0.064	0.245	0.478	0.068	0.036 *	0.555
miR-504-5p	7.36 ± 1.73	6.12 ± 1.15	8.22 ± 2.02	7.12 ± 0.99	7.00 ± 1.64	0.091	0.626	0.107	0.423	0.787	0.023 *	0.129	0.296

Data are presented as mean ± SD of ΔCt; a lower ΔCt indicates higher miRNA expression. * Significant at *p* < 0.05. PACS, primary angle-closure suspect; PAC, primary angle closure; PACG, primary angle-closure glaucoma; PACD, primary angle-closure disease.

**Table 3:** Correlation between miRNA expression (ΔCt) and anterior segment parameters.

Parameter	miR-146b-5p	miR-188-5p	miR-335-5p
max IOP	0.12 (0.561/0.678)	−0.22 (0.274/0.508)	0.14 (0.493/0.678)
AXL	0.10 (0.613/0.678)	0.41 (0.032/0.139) *	0.52 (0.005/0.084) *
CCT	−0.26 (0.190/0.443)	−0.08 (0.705/0.725)	−0.29 (0.140/0.368)
ACD	0.34 (0.085/0.255)	0.23 (0.251/0.508)	0.07 (0.725/0.725)
AOD500	0.11 (0.602/0.678)	0.50 (0.015/0.105) *	0.43 (0.042/0.147) *
Lens thickness	−0.16 (0.414/0.621)	−0.17 (0.395/0.621)	−0.12 (0.550/0.678)
lens vault	−0.75 (0.008/0.084) *	−0.64 (0.033/0.139) *	−0.35 (0.290/0.508)

Data are presented as Spearman correlation coefficient r (raw *p*-value/Benjamin–Hochberg FDR-adjusted *p*-value). ΔCt is inversely related to expression, so the sign of r is opposite to that for expression level. * Significant at *p* < 0.05. IOP, intraocular pressure; AXL, axial length; CCT, central corneal thickness; ACD, anterior chamber depth; AOD500, angle opening distance at 500 µm.

## Data Availability

The data supporting the findings of this study are not publicly available and cannot be shared due to privacy and ethical restrictions imposed by the institutional review board.

## Supplementary Materials

The following supporting information can be downloaded at: https://www.mdpi.com/article/10.3390/ijms27219556/s1.
