# Supplementary material for: Lens Capsule microRNA Profiling Across the Primary Angle-Closure Disease Spectrum: Association with Anterior Segment Crowding
Source: Int J Mol Sci. 2026 Oct 26;27(21):9556. doi: 10.3390/ijms27219556 (PMC13763522; doi:10.3390/ijms27219556)
Supplement: Supplementary file 1 [file ijms-27-09556-s001.zip › ijms-4629983-supplementary.pdf]

Supplementary Table S1. Differential expression (ΔCt) of all 84 analyzed miRNAs

miRNA	Control (Group 1)	PACS (Group 2)	PAC (Group 3)	PACG (Group 4)	PACD (Group 2-4)	Among Groups	Groups 1 vs. 2~4	Groups 1 vs 2	Groups 1 vs. 3	Groups 1 vs. 4	Groups 2 vs. 3	Groups 2 vs. 4	Groups 3 vs. 4
let-7a-5p	12.81±1.19	11.85±1.35	12.12±1.27	13.13±0.66	12.25±1.25	0.210	0.315	0.161	0.334	0.601	0.707	0.074	0.145
let-7b-5p	-0.34±1.29	-1.44±0.71	-1.34±0.48	-0.87±0.52	-1.27±0.62	0.074	0.109	**0.046***	0.093	0.412	0.770	0.141	0.149
let-7c-5p	-1.47±1.47	-2.34±0.96	-2.20±0.52	-1.71±0.75	-2.14±0.80	0.350	0.293	0.178	0.259	0.755	0.758	0.230	0.227
let-7d-5p	1.57±1.54	0.91±0.82	1.27±0.60	1.44±0.91	1.15±0.78	0.626	0.515	0.291	0.666	0.871	0.377	0.289	0.719
let-7e-5p	1.53±1.80	0.21±0.94	0.38±0.50	0.96±0.61	0.45±0.78	0.136	0.172	0.080	0.152	0.523	0.694	0.137	0.117
let-7f-5p	1.16±2.05	0.33±1.20	0.30±0.47	0.86±0.87	0.46±0.94	0.569	0.410	0.328	0.320	0.768	0.956	0.406	0.207
let-7g-5p	0.58±1.83	-0.39±1.07	-0.14±0.41	0.12±0.71	-0.19±0.82	0.443	0.320	0.205	0.349	0.606	0.536	0.367	0.476
let-7i-5p	2.48±2.06	1.33±0.75	1.37±0.50	1.57±0.52	1.41±0.61	0.247	0.219	0.201	0.211	0.298	0.914	0.545	0.534
miR-101-3p	1.60±2.34	0.86±1.41	1.31±0.49	1.02±1.39	1.03±1.16	0.811	0.557	0.442	0.755	0.634	0.397	0.836	0.682
miR-103a-3p	0.92±1.58	0.11±0.69	0.52±0.34	0.47±0.80	0.32±0.64	0.455	0.365	0.243	0.535	0.576	0.209	0.394	0.898
miR-107	1.64±1.78	0.52±0.67	1.02±0.34	0.99±0.88	0.79±0.67	0.257	0.257	0.156	0.396	0.469	0.123	0.287	0.939
miR-10b-5p	12.81±1.19	11.40±1.54	12.12±1.27	11.17±2.52	11.56±1.71	0.274	0.087	0.066	0.334	0.159	0.364	0.830	0.436
miR-122-5p	11.69±1.38	11.66±1.51	12.12±1.27	13.13±0.66	12.17±1.35	0.223	0.433	0.971	0.574	0.058	0.554	0.064	0.145
miR-125a-5p	-0.94±1.21	-1.36±0.95	-1.07±0.34	-0.91±0.65	-1.16±0.73	0.751	0.578	0.454	0.800	0.954	0.420	0.367	0.607
miR-125b-5p	-2.95±1.39	-3.38±1.06	-3.19±0.32	-2.97±0.73	-3.22±0.80	0.816	0.528	0.488	0.664	0.976	0.626	0.458	0.515
miR-130a-3p	0.55±1.99	-0.24±1.16	-0.15±0.20	-0.25±0.85	-0.22±0.86	0.604	0.357	0.334	0.388	0.424	0.828	0.995	0.821
miR-130b-3p	6.20±0.57	5.82±1.16	6.22±0.55	6.84±0.98	6.19±1.01	0.269	0.986	0.445	0.966	0.184	0.459	0.126	0.216
miR-135a-5p	6.53±2.61	4.97±1.87	4.23±2.41	5.28±2.07	4.83±2.02	0.324	0.087	0.186	0.130	0.396	0.513	0.784	0.464
miR-138-5p	8.98±2.16	8.98±2.06	9.69±2.11	7.58±0.74	8.84±1.93	0.363	0.877	0.999	0.560	0.199	0.525	0.174	0.063
miR-141-3p	6.97±3.36	5.95±2.30	5.96±0.47	5.72±1.83	5.89±1.73	0.767	0.444	0.483	0.460	0.470	0.990	0.850	0.786
miR-146a-5p	8.12±1.70	7.45±0.94	7.79±2.35	6.35±2.59	7.28±1.89	0.431	0.310	0.336	0.775	0.181	0.752	0.403	0.359
miR-146b-5p	6.91±1.88	5.03±1.03	5.59±0.97	5.88±0.85	5.41±0.99	0.056	0.083	**0.023***	0.150	0.285	0.317	0.145	0.608
miR-148a-3p	1.52±2.12	0.55±1.31	0.79±0.34	0.89±1.00	0.71±1.00	0.588	0.360	0.277	0.400	0.555	0.614	0.624	0.835
miR-148b-3p	3.67±1.65	2.93±0.79	3.15±0.23	3.23±0.92	3.07±0.69	0.578	0.385	0.255	0.443	0.607	0.446	0.530	0.858

miRNA	Control (Group 1)	PACS (Group 2)	PAC (Group 3)	PACG (Group 4)	PACD (Group 2-4)	Among Groups	Groups 1 vs. 2~4	Groups 1 vs 2	Groups 1 vs. 3	Groups 1 vs. 4	Groups 2 vs. 3	Groups 2 vs. 4	Groups 3 vs. 4
miR-150-5p	8.40±1.33	8.46±1.76	9.24±1.44	7.68±0.69	8.50±1.52	0.375	0.875	0.936	0.296	0.299	0.385	0.364	0.054
miR-155-5p	12.18±1.77	11.36±1.62	10.50±2.15	12.02±1.92	11.27±1.85	0.382	0.268	0.351	0.150	0.886	0.389	0.504	0.250
miR-15a-5p	6.17±2.48	5.37±1.69	5.23±0.57	5.21±1.40	5.29±1.31	0.714	0.398	0.454	0.363	0.458	0.823	0.866	0.982
miR-15b-5p	3.24±1.40	2.74±1.41	2.86±0.61	3.11±0.71	2.87±1.03	0.843	0.466	0.496	0.556	0.854	0.850	0.602	0.550
miR-16-5p	2.59±2.21	1.70±1.32	1.88±0.81	1.93±0.94	1.81±1.05	0.676	0.399	0.331	0.454	0.546	0.768	0.742	0.935
miR-17-5p	2.67±1.76	1.99±0.95	2.03±0.41	2.32±1.03	2.08±0.82	0.659	0.423	0.332	0.384	0.703	0.910	0.546	0.539
miR-181a-5p	0.60±1.74	-0.37±0.86	-0.01±0.60	0.30±1.00	-0.09±0.84	0.395	0.342	0.162	0.412	0.737	0.386	0.210	0.545
miR-181b-5p	2.82±1.51	2.04±0.61	2.30±0.78	2.54±0.67	2.24±0.67	0.450	0.362	0.236	0.466	0.708	0.473	0.180	0.607
miR-181c-5p	5.83±2.33	4.43±1.25	5.01±0.36	5.31±0.72	4.82±0.97	0.298	0.303	0.142	0.390	0.592	0.218	0.177	0.393
miR-184	-4.04±1.36	-4.77±0.90	-4.37±0.60	-4.10±0.45	-4.48±0.75	0.421	0.436	0.216	0.588	0.926	0.366	0.149	0.422
miR-186-5p	5.05±0.96	4.73±0.92	4.96±0.55	5.14±1.01	4.90±0.82	0.833	0.688	0.507	0.834	0.890	0.600	0.460	0.718
miR-188-5p	11.27±2.29	8.34±2.23	7.44±1.63	8.91±2.42	8.21±2.09	**0.022***	**0.003***	**0.022***	**0.006***	0.116	0.414	0.664	0.259
miR-191-5p	2.01±1.10	1.51±0.63	1.81±0.52	2.11±0.83	1.75±0.67	0.502	0.463	0.272	0.686	0.865	0.362	0.152	0.473
miR-192-5p	4.83±1.20	4.25±1.14	4.52±0.92	4.95±1.23	4.51±1.08	0.650	0.509	0.338	0.614	0.874	0.638	0.306	0.526
miR-195-5p	2.29±1.70	1.47±0.98	1.85±0.47	2.02±0.90	1.72±0.83	0.539	0.421	0.240	0.532	0.756	0.390	0.315	0.692
miR-199a-5p	8.29±2.46	8.44±2.57	7.02±1.31	9.24±3.40	8.21±2.54	0.528	0.948	0.905	0.283	0.584	0.237	0.628	0.172
miR-199b-5p	6.01±1.52	5.78±0.95	5.83±0.51	6.34±1.22	5.94±0.91	0.819	0.880	0.714	0.784	0.694	0.895	0.354	0.377
miR-19a-3p	1.55±2.03	0.64±1.22	0.82±0.46	1.03±1.19	0.79±1.00	0.603	0.370	0.278	0.382	0.620	0.695	0.566	0.687
miR-200a-3p	8.02±2.71	7.04±2.03	6.76±1.17	8.38±2.77	7.29±2.03	0.547	0.464	0.425	0.318	0.826	0.765	0.319	0.223
miR-200b-3p	8.04±2.53	6.79±1.46	7.04±1.36	7.55±0.66	7.05±1.25	0.508	0.354	0.232	0.404	0.638	0.746	0.297	0.464
miR-203a-3p	6.61±2.85	6.93±1.15	7.78±1.88	6.91±3.58	7.18±2.08	0.837	0.578	0.787	0.411	0.876	0.295	0.989	0.616
miR-204-5p	-3.89±1.28	-4.35±1.02	-4.15±0.45	-3.91±0.87	-4.18±0.83	0.770	0.494	0.433	0.624	0.983	0.664	0.425	0.555
miR-205-5p	3.77±1.83	4.55±1.12	5.83±2.61	3.94±2.55	4.78±2.06	0.284	0.262	0.308	0.124	0.893	0.300	0.634	0.259
miR-20a-5p	1.96±2.05	1.02±1.06	1.25±0.44	1.48±1.13	1.20±0.91	0.559	0.375	0.249	0.403	0.643	0.617	0.462	0.666
miR-20b-5p	9.88±3.06	9.70±2.83	11.46±1.12	10.09±2.55	10.33±2.38	0.606	0.696	0.907	0.242	0.904	0.177	0.806	0.263

miRNA	Control (Group 1)	PACS (Group 2)	PAC (Group 3)	PACG (Group 4)	PACD (Group 2-4)	Among Groups	Groups 1 vs. 2~4	Groups 1 vs 2	Groups 1 vs. 3	Groups 1 vs. 4	Groups 2 vs. 3	Groups 2 vs. 4	Groups 3 vs. 4
miR-210-3p	2.11±1.85	1.10±0.72	1.64±0.67	1.41±0.74	1.34±0.71	0.370	0.321	0.211	0.551	0.450	0.163	0.448	0.606
miR-215-5p	5.72±2.43	4.51±1.00	5.49±0.54	5.40±1.27	5.03±1.04	0.397	0.487	0.251	0.816	0.791	**0.048***	0.174	0.868
miR-21-5p	2.22±2.13	1.37±1.05	0.80±1.60	1.49±1.72	1.23±1.36	0.481	0.164	0.309	0.208	0.540	0.421	0.872	0.511
miR-221-3p	3.20±1.21	2.43±0.41	2.58±0.26	2.45±0.90	2.48±0.51	0.230	0.172	0.151	0.228	0.271	0.457	0.955	0.775
miR-22-3p	1.29±1.94	0.50±0.68	0.63±0.29	0.39±0.96	0.51±0.65	0.488	0.334	0.336	0.408	0.364	0.658	0.805	0.608
miR-224-5p	6.94±1.99	6.76±1.21	6.30±0.37	6.71±0.84	6.61±0.92	0.837	0.678	0.818	0.427	0.814	0.307	0.946	0.298
miR-23a-3p	-1.28±1.83	-2.11±0.94	-1.85±0.33	-1.76±0.91	-1.94±0.77	0.571	0.382	0.256	0.446	0.601	0.463	0.516	0.846
miR-23b-3p	-1.67±1.65	-2.68±0.99	-2.28±0.38	-2.17±0.88	-2.43±0.82	0.369	0.281	0.151	0.375	0.557	0.371	0.357	0.780
miR-24-3p	-2.32±1.65	-3.41±0.74	-3.07±0.38	-2.92±0.88	-3.19±0.69	0.240	0.220	0.142	0.284	0.477	0.311	0.286	0.723
miR-26a-5p	-1.33±1.83	-2.29±0.98	-2.06±0.44	-1.79±0.82	-2.10±0.80	0.432	0.317	0.196	0.337	0.615	0.606	0.349	0.489
miR-26b-5p	1.44±2.10	0.35±1.13	0.32±0.61	0.99±0.75	0.50±0.92	0.347	0.291	0.203	0.220	0.659	0.952	0.283	0.136
miR-27a-3p	-0.60±2.24	-1.79±1.25	-1.50±0.49	-1.32±1.12	-1.58±1.01	0.454	0.301	0.199	0.340	0.530	0.597	0.497	0.731
miR-29b-3p	2.09±2.63	1.06±1.00	0.96±0.38	1.01±1.21	1.02±0.88	0.492	0.325	0.354	0.300	0.414	0.795	0.935	0.936
miR-30b-5p	0.27±1.93	-0.43±1.30	-0.27±0.34	-0.17±1.08	-0.32±1.00	0.771	0.468	0.401	0.494	0.660	0.733	0.709	0.848
miR-30e-5p	2.89±1.71	2.08±1.11	2.43±0.50	2.36±1.21	2.26±0.96	0.633	0.233	0.268	0.518	0.569	0.483	0.665	0.903
miR-31-5p	0.49±1.57	-0.69±0.72	-0.19±0.50	0.02±1.11	-0.36±0.80	0.192	0.211	0.104	0.312	0.580	0.168	0.170	0.683
miR-320a-3p	0.79±1.22	0.34±0.58	0.52±0.51	0.76±0.65	0.50±0.57	0.671	0.565	0.345	0.632	0.958	0.539	0.239	0.523
miR-324-5p	5.05±0.76	4.84±0.94	5.30±0.46	4.80±0.52	4.97±0.73	0.628	0.811	0.646	0.495	0.546	0.292	0.929	0.125
miR-331-3p	4.00±1.71	3.20±1.06	3.51±0.56	3.56±0.97	3.39±0.88	0.615	0.390	0.267	0.496	0.618	0.523	0.542	0.916
miR-335-5p	7.15±3.16	4.42±1.06	5.55±1.09	6.03±1.48	5.16±1.32	0.065	0.151	0.064	0.245	0.478	0.068	**0.036***	0.555
miR-34a-5p	4.64±3.28	3.14±1.24	3.07±0.86	2.63±0.78	2.99±1.01	0.271	0.235	0.286	0.261	0.163	0.907	0.428	0.403
miR-378a-3p	2.47±1.82	1.80±0.75	2.50±0.53	1.99±0.85	2.06±0.75	0.557	0.578	0.390	0.972	0.596	0.073	0.681	0.253
miR-429	8.81±2.11	8.34±2.25	9.26±1.38	8.47±2.70	8.65±2.08	0.862	0.861	0.677	0.663	0.809	0.389	0.927	0.542
miR-449a	12.81±1.19	10.65±2.59	12.12±1.27	12.23±2.10	11.48±2.19	0.177	0.143	0.061	0.334	0.554	0.223	0.268	0.917
miR-451a	4.92±4.21	4.10±5.11	5.13±3.51	3.91±3.15	4.36±4.07	0.943	0.759	0.736	0.923	0.660	0.673	0.941	0.560

miRNA	Control (Group 1)	PACS (Group 2)	PAC (Group 3)	PACG (Group 4)	PACD (Group 2-4)	Among Groups	Groups 1 vs. 2~4	Groups 1 vs 2	Groups 1 vs. 3	Groups 1 vs. 4	Groups 2 vs. 3	Groups 2 vs. 4	Groups 3 vs. 4
miR-491-5p	5.94±0.47	5.24±1.07	5.85±1.10	5.57±0.82	5.51±1.00	0.441	0.283	0.130	0.837	0.340	0.308	0.563	0.656
miR-504-5p	7.36±1.73	6.12±1.15	8.22±2.02	7.12±0.99	7.00±1.64	0.091	0.626	0.107	0.423	0.787	**0.023***	0.129	0.296
miR-7-5p	9.08±1.85	9.05±2.08	8.44±2.40	10.19±2.49	9.15±2.26	0.618	0.943	0.974	0.596	0.395	0.608	0.375	0.266
miR-877-3p	8.54±1.58	8.40±1.98	7.94±1.86	7.55±1.33	8.05±1.75	0.756	0.519	0.880	0.542	0.282	0.660	0.411	0.705
miR-92a-3p	1.14±1.03	0.70±0.73	0.83±0.48	1.18±0.67	0.86±0.65	0.583	0.409	0.333	0.514	0.931	0.708	0.242	0.329
miR-935	3.55±1.08	3.41±1.12	3.61±0.75	4.00±0.87	3.62±0.94	0.766	0.869	0.810	0.905	0.458	0.708	0.330	0.447
miR-93-5p	2.84±1.72	1.98±0.72	2.33±0.50	2.57±0.72	2.23±0.68	0.428	0.393	0.248	0.481	0.751	0.308	0.162	0.537
miR-9-5p	6.98±2.19	6.53±2.48	7.21±2.07	8.39±2.83	7.20±2.45	0.585	0.834	0.713	0.847	0.352	0.589	0.226	0.447
miR-98-5p	4.95±0.97	4.18±0.88	4.20±0.65	4.88±0.66	4.36±0.79	0.184	0.123	0.120	0.138	0.899	0.965	0.148	0.119
miR-99a-5p	-1.16±1.83	-1.76±1.09	-1.50±0.65	-1.11±0.90	-1.52±0.92	0.720	0.630	0.424	0.656	0.959	0.609	0.279	0.425

Data are mean ± SD of ΔCt; a lower ΔCt indicates higher miRNA expression. *Significant at $p < 0.05$.

PACS, primary angle closure suspect; PAC, primary angle closure; PACG, primary angle closure glaucoma; PACD, primary angle closure disease.

Supplementary Table S2. Differential expression (ΔCt) of all 84 analyzed miRNAs between eyes with and without a history of acute angle-closure attack, within PACD group.

miRNA	Non-attack (n=10) ΔCt, Mean ± SD	Attack (n=10) ΔCt, Mean ± SD	p value
hsa-let-7a-5p	12.05 ± 1.29	12.45 ± 1.25	0.488
hsa-let-7b-5p	-1.36 ± 0.69	-1.18 ± 0.57	0.519
hsa-let-7c-5p	-2.27 ± 0.89	-2.01 ± 0.72	0.481
hsa-let-7d-5p	0.98 ± 0.78	1.33 ± 0.78	0.331
hsa-let-7e-5p	0.33 ± 0.90	0.57 ± 0.68	0.512
hsa-let-7f-5p	0.42 ± 1.12	0.49 ± 0.77	0.874
hsa-let-7g-5p	-0.30 ± 1.00	-0.07 ± 0.63	0.558
hsa-let-7i-5p	1.43 ± 0.72	1.39 ± 0.52	0.891
hsa-miR-101-3p	0.95 ± 1.34	1.11 ± 1.01	0.764
hsa-miR-103a-3p	0.18 ± 0.67	0.46 ± 0.61	0.350
hsa-miR-107	0.60 ± 0.65	0.98 ± 0.66	0.209
hsa-miR-10b-5p	11.19 ± 1.88	11.93 ± 1.52	0.343
hsa-miR-122-5p	11.88 ± 1.46	12.45 ± 1.25	0.360
hsa-miR-125a-5p	-1.23 ± 0.89	-1.08 ± 0.57	0.661
hsa-miR-125b-5p	-3.30 ± 1.01	-3.14 ± 0.57	0.668
hsa-miR-130a-3p	-0.21 ± 1.10	-0.22 ± 0.58	0.970
hsa-miR-130b-3p	5.96 ± 1.13	6.42 ± 0.87	0.320
hsa-miR-135a-5p	5.25 ± 2.06	4.41 ± 1.99	0.367
hsa-miR-138-5p	8.35 ± 1.92	9.33 ± 1.91	0.265
hsa-miR-141-3p	6.12 ± 2.16	5.67 ± 1.24	0.577
hsa-miR-146a-5p	7.19 ± 0.83	7.37 ± 2.61	0.840
hsa-miR-146b-5p	5.10 ± 1.01	5.72 ± 0.92	0.171
hsa-miR-148a-3p	0.65 ± 1.26	0.76 ± 0.71	0.802
hsa-miR-148b-3p	3.06 ± 0.78	3.08 ± 0.62	0.963
hsa-miR-150-5p	7.99 ± 1.36	9.01 ± 1.56	0.134
hsa-miR-155-5p	11.61 ± 1.60	10.93 ± 2.10	0.425
hsa-miR-15a-5p	5.33 ± 1.66	5.24 ± 0.92	0.884
hsa-miR-15b-5p	2.85 ± 1.30	2.89 ± 0.73	0.934
hsa-miR-16-5p	1.70 ± 1.26	1.92 ± 0.85	0.654
hsa-miR-17-5p	2.03 ± 0.90	2.14 ± 0.76	0.774
hsa-miR-181a-5p	-0.25 ± 0.88	0.07 ± 0.81	0.404
hsa-miR-181b-5p	2.16 ± 0.59	2.32 ± 0.77	0.609
hsa-miR-181c-5p	4.73 ± 1.26	4.92 ± 0.63	0.681
hsa-miR-184	-4.64 ± 0.86	-4.32 ± 0.61	0.344
hsa-miR-186-5p	4.73 ± 0.86	5.07 ± 0.79	0.370
hsa-miR-188-5p	8.50 ± 1.94	7.92 ± 2.29	0.549
hsa-miR-191-5p	1.58 ± 0.64	1.92 ± 0.69	0.259
hsa-miR-192-5p	4.45 ± 1.10	4.57 ± 1.12	0.816
hsa-miR-195-5p	1.60 ± 0.97	1.85 ± 0.70	0.517
hsa-miR-199a-5p	8.45 ± 2.64	7.98 ± 2.54	0.688
hsa-miR-199b-5p	5.91 ± 0.98	5.97 ± 0.88	0.887
hsa-miR-19a-3p	0.74 ± 1.17	0.84 ± 0.86	0.840
hsa-miR-200a-3p	7.28 ± 1.88	7.30 ± 2.28	0.979
hsa-miR-200b-3p	7.05 ± 1.33	7.06 ± 1.24	0.986
hsa-miR-203a-3p	7.29 ± 2.28	7.07 ± 1.98	0.815
hsa-miR-204-5p	-4.29 ± 0.98	-4.07 ± 0.68	0.563

miRNA	Non-attack (n=10) ΔCt, Mean ± SD	Attack (n=10) ΔCt, Mean ± SD	p value
hsa-miR-205-5p	4.74 ± 1.11	4.82 ± 2.78	0.937
hsa-miR-20a-5p	1.09 ± 1.01	1.32 ± 0.83	0.586
hsa-miR-20b-5p	10.12 ± 2.68	10.53 ± 2.17	0.712
hsa-miR-210-3p	1.18 ± 0.70	1.50 ± 0.72	0.332
hsa-miR-215-5p	4.65 ± 0.99	5.40 ± 0.99	0.108
hsa-miR-21-5p	1.47 ± 1.00	0.98 ± 1.66	0.429
hsa-miR-221-3p	2.43 ± 0.40	2.53 ± 0.62	0.682
hsa-miR-22-3p	0.53 ± 0.65	0.49 ± 0.68	0.887
hsa-miR-224-5p	6.78 ± 1.15	6.44 ± 0.63	0.421
hsa-miR-23a-3p	-2.03 ± 0.91	-1.86 ± 0.65	0.634
hsa-miR-23b-3p	-2.56 ± 0.95	-2.30 ± 0.68	0.484
hsa-miR-24-3p	-3.29 ± 0.75	-3.08 ± 0.65	0.514
hsa-miR-26a-5p	-2.16 ± 0.94	-2.04 ± 0.68	0.752
hsa-miR-26b-5p	0.46 ± 1.07	0.54 ± 0.79	0.855
hsa-miR-27a-3p	-1.65 ± 1.20	-1.52 ± 0.84	0.786
hsa-miR-29b-3p	1.12 ± 0.98	0.91 ± 0.80	0.621
hsa-miR-30b-5p	-0.36 ± 1.25	-0.27 ± 0.74	0.855
hsa-miR-30e-5p	2.11 ± 1.07	2.40 ± 0.87	0.524
hsa-miR-31-5p	-0.54 ± 0.79	-0.18 ± 0.81	0.331
hsa-miR-320a-3p	0.41 ± 0.57	0.59 ± 0.60	0.510
hsa-miR-324-5p	4.93 ± 0.91	5.01 ± 0.54	0.819
hsa-miR-331-3p	3.33 ± 1.00	3.44 ± 0.80	0.802
hsa-miR-335-5p	4.79 ± 1.30	5.54 ± 1.30	0.216
hsa-miR-34a-5p	3.05 ± 1.23	2.93 ± 0.79	0.807
hsa-miR-378a-3p	1.84 ± 0.73	2.28 ± 0.74	0.194
hsa-miR-429	8.38 ± 1.94	8.92 ± 2.29	0.579
hsa-miR-449a	11.21 ± 2.46	11.76 ± 1.97	0.591
hsa-miR-451a	4.11 ± 5.02	4.61 ± 3.11	0.792
hsa-miR-491-5p	5.31 ± 1.09	5.70 ± 0.92	0.403
hsa-miR-504-5p	6.43 ± 1.29	7.58 ± 1.81	0.118
hsa-miR-7-5p	9.53 ± 2.09	8.77 ± 2.46	0.467
hsa-miR-877-3p	8.25 ± 1.97	7.85 ± 1.57	0.621
hsa-miR-92a-3p	0.74 ± 0.70	0.97 ± 0.60	0.440
hsa-miR-935	3.46 ± 1.05	3.78 ± 0.85	0.465
hsa-miR-93-5p	2.09 ± 0.71	2.38 ± 0.65	0.360
hsa-miR-9-5p	7.09 ± 2.01	7.31 ± 2.93	0.850
hsa-miR-98-5p	4.33 ± 0.82	4.39 ± 0.81	0.875
hsa-miR-99a-5p	-1.65 ± 1.05	-1.39 ± 0.81	0.548

Data are presented as mean ± SD of ΔCt; a lower ΔCt indicates higher miRNA expression. *Significant at $P > 0.05$.
